# Research advances in Janus kinase inhibitors for immune checkpoint inhibitors-related myocarditis

**DOI:** 10.3389/fimmu.2026.1857298

**Published:** 2026-10-20

**Authors:** Jing Leng, Chenyu Wang, Chunmei Bai

**Affiliations:** Department of Medical Oncology, Peking Union Medical College Hospital, Chinese Academy of Medical Sciences, Beijing, China

**Keywords:** immune checkpoint inhibitor-related myocarditis, immune-related adverse events, JAK inhibitors, ruxolitinib, tofacitinib

## Abstract

Immune checkpoint inhibitor-related myocarditis (ICIM) is a rare yet potentially lethal complication of immune checkpoint inhibitor (ICI) therapy, which severely impacts patient prognosis and therapeutic efficacy. With the widespread application of immunotherapy, ICIM has attracted significant attention from clinicians and researchers. However, its underlying pathogenesis remains to be fully elucidated, its clinical manifestations lack specificity, and management is frequently hampered by a dearth of effective, safe interventions. The Janus kinase (JAK) signaling pathway plays a critical role in regulating immune and inflammatory responses. JAK inhibitors exert immunomodulatory effects by blocking the JAK-STAT signaling pathway and have been utilized to treat a spectrum of immune-mediated disorders, emerging as a promising therapeutic strategy for ICIM. Accumulating evidence suggests that JAK inhibition may improve clinical outcomes in patients with ICIM. This review summarizes the latest advancements in JAK inhibitors for ICIM, aiming to provide a theoretical foundation and guide the optimization of future therapeutic strategies and clinical decisions.

## 1 Introduction

Immune checkpoint inhibitors (ICIs) have revolutionized cancer immunotherapy, substantially improving survival in patients with various malignancies. By antagonizing the programmed death-1 (PD-1)/programmed death-ligand 1 (PD-L1) and cytotoxic T-lymphocyte antigen-4 (CTLA-4) pathways, ICIs release T cells from inhibitory signaling and enhance T-cell-mediated anti-tumor immune responses (1). However, the burgeoning clinical utility of ICIs has been accompanied by the emergence of distinct limitations. Specifically, systemic immune overactivation can trigger off-target effects, thereby escalating the risk of severe immune-related adverse events (irAEs) (2). IrAEs encompass systemic multi-organ involvement, with cardiovascular manifestations including Immune checkpoint inhibitors-related myocarditis (ICIM), pericarditis, and atherosclerotic cardiovascular disease. While ICIM occurs infrequently, its exceptionally high lethality renders it a formidable complication requiring prioritized recognition and management (3). Mechanistically, the removal of checkpoint inhibition by ICIs leads to dysregulated T-cell responses that target the myocardium, causing profound inflammation and injury (4, 5). This process is characterized by CD8+ T-cell infiltration and cytokine release, driven largely by the activation of inflammatory pathways—most notably the Janus kinase-signal transducer and activator of transcription (JAK-STAT) axis (6). Despite the lack of a clinical consensus on its exact mechanisms, preclinical studies have highlighted the pathogenic role of JAK-STAT signaling in ICIM (7, 8).

Currently, glucocorticoids remain the cornerstone of first-line therapy for ICIM. For steroid-resistant patients, adjunctive or alternative immunosuppressants are employed, such as tacrolimus, mycophenolate mofetil, or intravenous immunoglobulin (IVIG), though clinical outcomes remain constrained (9–11). As emerging immunomodulatory agents, the Janus kinase (JAK) inhibitors specifically antagonize the JAK-STAT signaling pathway, exerting precise control over immune cell activation and inflammatory cascades. They demonstrate therapeutic potency across a broad spectrum of autoimmune disorders (12). Emerging case reports and clinical evidence suggest that JAK inhibition effectively manages irAEs. Nevertheless, international guidelines have yet to formally endorse JAK inhibitors as a standard of care for ICIM. Therefore, this targeted approach holds the potential to improve the management of ICI-associated cardiotoxicity.

We conducted a narrative review for this work. Literature retrieval was performed via PubMed and Embase databases up to December 2025, with search terms covering “immune checkpoint inhibitor-related myocarditis”, “ICIM”, “JAK inhibitor”, “tofacitinib”, “ruxolitinib”, “baricitinib”, and “immune-related adverse event”. We also manually screened reference lists of included original studies and reviews to identify extra relevant publications. While multiple existing reviews have summarized the broad application of JAK inhibitors across various immune-related adverse events, the present review integrates the latest clinical evidence of JAK-inhibitor therapy specifically for ICIM, and extends the discussion to future research directions such as biomarker-driven patient stratification and predictive response modeling.

## 2 Pathogenesis, clinical manifestations, and treatment of ICIM

The pathogenesis of ICIM primarily involves the aberrant activation of the immune system. While the precise pathophysiological mechanisms underlying irAEs remain to be fully elucidated, several interconnected pathways have been proposed: First, ICIs bolster the activation and proliferation of T cells while concurrently compromising peripheral T-cell tolerance, thereby precipitating hyperinflammatory and autoimmune responses (13, 14). Second, the surge of cytokines, such as IFN-γ, tumor necrosis factor-α (TNF-α), and other pro-inflammatory mediators, bind to immune cell receptors to trigger intracellular cascades—notably the JAK-STAT and PI3K-AKT-mTOR pathways—resulting in a profound dysregulation of the pro-inflammatory response (14–16). Furthermore, the production of autoantibodies in a subset of patients may exacerbate immune-mediated tissue destruction.

Specifically in ICIM, the antagonism of PD-1 and CTLA-4 axes unleashes autoreactive T cells. Most notably, CD8+ cytotoxic T cells specifically targeting the antigen α-myosin are activated, leading to a direct assault on cardiomyocytes and subsequent myocardial injury (17, 18). During the pathogenesis of Immune Checkpoint Inhibitor-associated Myocarditis (ICIM), aberrant T-cell hyperactivation triggers the IL-6/STAT3 signaling axis. Elevated systemic IL-6 levels engage the JAK1/JAK2/TYK2 complex, subsequently inducing the tyrosine phosphorylation and activation of STAT3. Concurrently, activated T cells secrete IFN-γ, which predominantly signals through the JAK1/JAK2-STAT1 pathway while also contributing to STAT3 activation. This IFN-γ-rich milieu promotes macrophage polarization and the localized secretion of the chemokines CXCL9 and CXCL10, which orchestrate the recruitment of additional effector T cells into the myocardium. This cascade establishes a pro-inflammatory positive feedback loop, progressively exacerbating myocardial injury (19, 20). The terminal pathological manifestation of myocarditis is myocardial fibrosis (21). Histologically, ICIM is characterized by patchy myocardial leukocytic infiltration, with pathological hallmarks predominantly involving CD8+ T-cell-centered lymphocytic infiltration and extensive myocyte damage (22). Single-cell transcriptomic profiling has revealed the presence of pro-inflammatory cellular subsets, such as CXCL9+ fibroblasts and ACKR1+ endothelial cells, within the myocardial tissues of patients with ICI-associated myocarditis. These subsets drive inflammatory amplification and immune cell recruitment via the JAK-STAT and TNF-α signaling pathways, thereby orchestrating a distinct inflammatory microenvironment (6). Consistently, RNA sequencing data from ICIM murine models demonstrate a significant upregulation of JAK2 and the broader JAK-STAT signaling axis compared to controls, suggesting that the dysregulation of this pathway drives the observed inflammatory cascade and structural damage (7, 23).

Most immune-related adverse events (irAEs) affect various organ systems and carry a relatively low mortality rate. However, ICIM is associated with the highest fatality rate among all irAEs, with mortality estimates between 38% and 50%, notwithstanding its relatively rare incidence of 0.1%–2% (24–27). Clinically, ICIM presents with a heterogeneous spectrum of manifestations, including arrhythmias, heart failure, and cardiogenic shock. Patients may exhibit non-specific symptoms such as chest pain, palpitations, fatigue, and dyspnea, or remain entirely asymptomatic. Electrocardiographic (ECG) manifestations may include ST-segment elevation, arrhythmias, or conduction blocks; notably, certain cases mimic acute coronary syndrome, thereby complicating the diagnostic process (28). Regarding biomarkers, cardiac troponin I (cTnI) remains the most reliable early predictor of severe myocarditis. Elevated levels of cTnI, creatine kinase-MB (CK-MB), and brain natriuretic peptide (BNP) serve as essential indicators for monitoring cardiotoxicity and supporting initial diagnosis in symptomatic patients, although their diagnostic sensitivity and specificity are notably limited (29, 30). Imaging modalities encompass echocardiography, cardiac magnetic resonance (CMR), and coronary angiography. In particular, CMR provides critical diagnostic evidence by enabling the assessment of myocardial inflammation and fibrosis (6, 31). Endomyocardial biopsy remains the gold standard for definitive diagnosis, especially when CMR or other imaging findings are inconclusive, or when immunophenotypic confirmation is required to guide targeted therapy. However, its clinical utility is hampered by its invasive nature and suboptimal sensitivity (32). To date, a unified international diagnostic standard for ICIM has not been established; current clinical practice largely relies on the 2022 International Cardio-Oncology Society (ICOS) consensus criteria (**Supplementary Table 1**). Furthermore, ICIM frequently co-occurs with multi-organ irAEs; approximately 23%–37% of patients present with concurrent myositis, while 11% manifest as myasthenia gravis. This overlap significantly amplifies clinical heterogeneity and diagnostic complexity (13, 33, 34). Retrospective evidence suggests that isolated ICIM is more often characterized by arrhythmias, whereas the concomitant myositis phenotype is predominantly associated with heart failure (35). Consequently, timely recognition and intervention are paramount, as diagnostic delays often culminate in fulminant heart failure and excessive mortality.

Given the marked clinical heterogeneity of ICIM, a standardized treatment consensus remains elusive. While stratified therapeutic approaches are increasingly recognized, their specific protocols await more robust, evidence-based support. Although the CTCAE Version 5.0 classifies myocarditis into a 5-grade system, ASCO and NCCN guidelines simplify this framework into two tiers: mild-to-moderate (G1–G2), featuring preserved cardiac function (LVEF ≥ 50%) without hemodynamic compromise, and severe (G3–G4), presenting as LVEF < 50%, malignant arrhythmia, or hemodynamic instability, which dictates the mandatory and permanent cessation of ICI therapy. Nonetheless, the prompt administration of high-dose glucocorticoids is paramount upon clinical suspicion or diagnosis. Notably, initiating corticosteroid therapy within 24 hours of admission has been associated with a substantially lower Major Adverse Cardiovascular Events (MACE) rate (7.0%) compared to delayed intervention beyond 24–72 hours (34.3%) (36). Such therapy should be rigorously maintained until the normalization of troponin levels, resolution of ECG anomalies, and restoration of left ventricular systolic function. For asymptomatic or mildly symptomatic immune checkpoint inhibitor-associated myocarditis (ICIM, Grades 1–2), glucocorticoids represent the well-established front-line therapeutic option. The recommended initial regimen is oral prednisone at 1–2 mg/kg/day, with a maximum dose capped at 80 mg/day, maintained for 2–4 weeks. Conversely, upon the diagnosis of fulminant ICIM (Grades 3–4), immediate ICI cessation and ICU admission are mandatory to initiate advanced life support interventions, including hemodynamic monitoring, mechanical ventilation, and temporary cardiac pacing. High-dose methylprednisolone pulse therapy is strongly recommended at 500–1000 mg/day intravenously for 3–5 days, followed by a transition to oral prednisone at 1–2 mg/kg/day with a subsequent gradual taper (37, 38). Concomitant myocarditis-myositis-myasthenia gravis (MG) overlap syndrome significantly exacerbates therapeutic complexity. Given that the immunopathology of myositis and MG overlaps with, yet diverges from, that of myocarditis, traditional monotherapeutic immunosuppression may be insufficient to achieve optimal inflammatory control across these distinct target organs. When conventional glucocorticoids prove suboptimal, the substitution or adjunctive use of immunosuppressants becomes a critical therapeutic maneuver. Calcineurin inhibitors, such as cyclosporine and tacrolimus, mitigate the myocardial immune response by suppressing T-cell activation and cytokine production. Calcineurin inhibitors like cyclosporine and tacrolimus inhibit T-cell activation and cytokine production, thereby mitigating the immune response in myocarditis. The anti-CD20 monoclonal antibody rituximab targets B cells to reduce autoantibody levels, alleviating immune inflammation. Intravenous immunoglobulin (IVIG), an immunomodulator, can help restore immune balance and modulate immune responses (39). Furthermore, the anti-interleukin-6 (IL-6) receptor inhibitor tocilizumab, which hampers IL-6 signaling and cytokine release, has demonstrated efficacy in patients refractory to steroids and conventional immunosuppressants (40, 41). A retrospective analysis identified TNF inhibitors (primarily infliximab, 17%), mycophenolate mofetil (10%), tacrolimus (4%), and IVIG (3%) as the most frequently utilized second-line immunomodulators (42). In the management of severe or corticosteroid-resistant neurological irAEs, escalating to second-line modalities, including IVIG or plasmapheresis, can confer cumulative clinical efficacy. Conventional therapies often lack precision in targeting specific immunopathological pathways, leaving significant room for optimization in both efficacy and safety profiles. There is an urgent exigency to develop novel targeted interventions, such as JAK inhibitors, to explore more precise, efficacious, and less toxic therapeutic strategies to improve patient outcomes.

## 3 Clinical evidence for JAK inhibitors in the treatment of ICIM

### 3.1 The JAK-STAT signaling pathway

The Janus kinase (JAK)/signal transducer and activator of transcription (STAT) signaling pathway represents a critical signal transduction mechanism regulated by a diverse array of growth factors and cytokines. This canonical pathway comprises tyrosine kinase-associated receptors, Janus kinases, and STAT proteins (43). The JAK family, a class of non-receptor tyrosine kinases, consists of four distinct members—JAK1, JAK2, JAK3, and Tyk2—which primarily mediate signal transduction from cytokine receptors. Concurrently, the STAT family encompasses seven members: STAT1, STAT2, STAT3, STAT4, STAT5a, STAT5b, and STAT6. Upon activation, JAKs recruit and phosphorylate specific STAT proteins (44, 45). Following the binding of cytokine ligands to their cognate receptors, JAK proteins undergo activation and auto-phosphorylation, subsequently triggering the activation of STAT proteins. These activated STATs form functional dimers that translocate into the nucleus to modulate the transcriptional expression of target genes, thereby orchestrating cellular processes such as proliferation, differentiation, apoptosis, and immune responses. Pro-inflammatory cytokines, including interleukin-6 (IL-6) and interferon-gamma (IFN-γ), utilize this pathway to exert their signaling functions in inflammatory and immunoregulatory contexts. Importantly, dysregulation of these signaling cascades is intricately linked to the pathogenesis of various immune-mediated diseases (46, 47).

Myocarditis is characterized by inflammatory cell infiltration into the myocardium, with the activation of inflammatory cascades serving as a hallmark of its pathogenesis (48). Pro-inflammatory cytokines, such as IL-6 and IFN-γ, promote the production of downstream inflammatory mediators and the recruitment of immune cells via the JAK-STAT and TNF-α signaling pathways. Notably, the IL-6-JAK-STAT3 axis plays a pivotal role in the inflammatory milieu of immune checkpoint inhibitor-induced myocarditis (ICIM). Clinical evidence further reveals that elevated serum IL-6 levels in myocarditis patients are closely associated with the aberrant activation of the JAK-STAT pathway, and its signaling intensity correlates positively with disease severity (23, 49). Increased expression of phosphorylated proteins within the JAK-STAT pathway has been observed in rat models of experimental autoimmune myocarditis (EAM). Notably, pharmacological intervention with JAK inhibitors has been shown to attenuate myocardial inflammation and ameliorate cardiac dysfunction (8). Consequently, the JAK signaling pathway plays a fundamental role in the inflammatory response and pathological progression of myocarditis by mediating pro-inflammatory cytokine signaling and immune cell activation. Thus, targeting the JAK cascade represents a promising therapeutic avenue for disease management.

### 3.2 JAK inhibitors

Janus kinase (JAK) inhibitors constitute a class of small-molecule agents that exert immunomodulatory effects by disrupting the JAK-STAT signaling cascade, with broad clinical utility across diverse immune-mediated pathologies. These inhibitors function through competitive binding to the ATP-binding pocket of the JAK enzymes, thereby precluding ATP association and abrogating catalytic kinase activity. This molecular blockade effectively halts signal transduction, preventing the phosphorylation and subsequent nuclear translocation of STAT proteins, which ultimately modulates downstream gene transcription (50). To date, numerous JAK inhibitors have received regulatory approval (**Table 1**). JAK inhibitors are categorized into pan-JAK, selective JAK1, and TYK2 inhibitors according to their isoform selectivity profiles (JAK1, JAK2, JAK3, and TYK2). First-generation pan-JAK inhibitors, including tofacitinib, peficitinib, and ruxolitinib, deliver robust anti-inflammatory activity via the simultaneous, non-selective inhibition of multiple JAK subunits. Nevertheless, this multi-target suppression may compromise endogenous cardioprotective pathways, elevating cardiomyocyte vulnerability to cellular damage and posing critical safety concerns, particularly within the cardiovascular domain. Tofacitinib, the pioneering small-molecule JAK inhibitor, targets JAK1, JAK2, JAK3, and TYK2 to modulate signaling pathways involving IL-6 and type I interferons; it is established in the treatment of rheumatoid arthritis, psoriatic arthritis, and ulcerative colitis (51, 52). Ruxolitinib modulates immune responses via orthosteric inhibition of the JAK1/2 ATP-binding sites and is approved for myelofibrosis and atopic dermatitis, with ongoing exploration of its off-label therapeutic potential (53). In the ORAL Surveillance trial, a mandatory post-marketing safety study in rheumatoid arthritis, tofacitinib—particularly within the high-dose cohort of 10 mg twice daily—demonstrated a significantly elevated risk of major adverse cardiovascular events (MACE) and venous thromboembolism (VTE) compared with tumor necrosis factor inhibitors (TNFi). These critical safety findings directly prompted the US Food and Drug Administration (FDA) to implement a boxed warning for this class of therapeutics (54). -In contrast, second-generation inhibitors, such as upadacitinib and abrocitinib, possess enhanced isoform specificity, frequently demonstrating superior selectivity for JAK1. Mechanistically, selective JAK1 inhibitors theoretically mitigate microvascular complications and alleviate JAK2-mediated hematological toxicities, such as anemia and neutropenia, by sparing potent JAK2 inhibition (55). However, real-world data derived from rheumatoid arthritis cohorts demonstrate that the risk of discontinuation due to adverse events does not differ significantly between selective JAK1 inhibitors and pan-JAK formulations (56). Furthermore, highly selective TYK2 inhibitors and their allosteric counterparts—particularly novel inhibitors targeting the pseudokinase (JH2) domain—are increasingly recognized as third-generation inhibitors. Deucravacitinib, a first-in-class allosteric TYK2 inhibitor, achieves exceptional selectivity for TYK2 by binding to its regulatory pseudokinase (JH2) domain rather than the highly conserved ATP-binding pocket, thereby minimizing off-target effects on JAK1–3 (57). This distinct mechanism of action theoretically translates into a lower propensity for cardiovascular toxicities. In landmark phase III clinical trials for psoriasis (POETYK PSO-1 and PSO-2), deucravacitinib demonstrated superior efficacy compared to both apremilast and placebo, coupled with a favorable safety profile and no discernible signals of severe adverse cardiovascular events typically associated with pan-JAK inhibitors (58). Currently, a pipeline of next-generation selective inhibitors is undergoing clinical evaluation to optimize efficacy and safety profiles. Collectively, the robust immunomodulatory properties of these JAK inhibitors provide a compelling theoretical framework for their application in managing immune checkpoint inhibitor-induced myocarditis (ICIM).

**Table 1:** JAK inhibitors approved by the national medical products administration (as of October 2025) with corresponding targets and indications.

Drug name (generic)	Primary target(s)	Approved indications in China (some include off-label uses)	Standard dosage	Year of approval/launch in China
Tofacitinib	JAK1, JAK3	Rheumatoid arthritis, Psoriatic arthritis, Ankylosing spondylitis, Ulcerative colitis	5 mg twice daily (immediate-release); 11 mg once daily (extended-release)	2017
Baricitinib	JAK1, JAK2	Rheumatoid arthritis, Moderate-to-severe atopic dermatitis	2 mg or 4 mg once daily	2019
Ruxolitinib (Oral)	JAK1, JAK2	Intermediate-or high-risk myelofibrosis, Polycythemia vera, Graft-versus-host disease	Based on platelet count, typically 5–25 mg twice daily	2017
Ruxolitinib (Topical Cream)	JAK1, JAK2	Atopic dermatitis, Non-segmental vitiligo	Topical application, twice daily	2023
Peficitinib	JAK3	Rheumatoid arthritis	400 mg twice daily	2021
Upadacitinib	JAK1	Rheumatoid arthritis, Psoriatic arthritis, Ankylosing spondylitis, Moderate-to-severe atopic dermatitis, Moderate-to-severe active ulcerative colitis	15 mg once daily	2022
Abrocitinib	JAK1	Atopic dermatitis	100 mg or 200 mg once daily	2022
Deucravacitinib	TYK2	Psoriasis	6 mg once daily	2023
Golidocitinib	JAK1	Relapsed or refractory peripheral T-cell lymphoma	150 mg once daily	2024
Ivarmacitinib Sulfate	JAK1	Ankylosing spondylitis	25 mg or 50 mg once daily	2024

### 3.3 Analysis of clinical studies and case reports on JAK inhibitor treatment for ICIM

In recent years, a growing body of clinical evidence has underscored the therapeutic potential of JAK inhibitors in managing irAEs, particularly ICIM (**Table 2**). Numerous case reports have documented the successful deployment of ruxolitinib and tofacitinib in ICIM (60, 63, 66). In a 2025 prospective study investigating JAK inhibitors (primarily tofacitinib) as a second-line or later-line treatments, a 70.8% complete remission rate of irAEs was achieved. Notably, this included resolution in 6 out of 8 patients with myocarditis. Furthermore, no opportunistic infections or JAK inhibitor-related hepatotoxicity or cardiotoxicity were observed in this cohort (65). A published narrative review synthesized real-world data from multiple published case series and observational cohorts, summarizing 104 patients with immune-related adverse events; myocarditis constituted 70% of these compiled cases, with tofacitinib (82%) and ruxolitinib (13%) being the most frequently applied agents (12). Data from a multicenter observational study highlighted the superior therapeutic efficacy of tofacitinib for irAEs, manifesting a remission rate of 54.5%–100% in steroid-refractory cohorts. Notably, an 87.5% remission rate was achieved specifically in ICIM cases, supported by a manageable safety profile with minimal infectious complications (64). Consistent with these findings, Wang et al. demonstrated that tofacitinib serves as a viable salvage intervention for steroid-resistant patients. Seven out of 11 steroid-refractory ICIM patients achieved complete clinical resolution under tofacitinib 5 mg twice-daily therapy (59).

**Table 2:** Summary of clinical reports on JAK inhibitors for the treatment of immune checkpoint inhibitor-related myocarditis.

Source	Article title	Study design	Number of cases	JAK inhibitor used (dosage)	Myocarditis outcome
(59)	Case Series of Steroid-Resistant Immune Checkpoint Inhibitor Associated Myocarditis: A Comparative Analysis of Corticosteroid and Tofacitinib Treatment	Retrospective Case Series	11	Tofacitinib (5 mg twice daily)	7 corticosteroid-resistant patients achieved complete recovery from ICIM.
(60)	Case Report: Treatment for steroid-refractory immune-related myocarditis with tofacitinib	Case Report	1	Tofacitinib (5 mg twice daily for 1 week)	Patient recovered with no major adverse cardiac events during hospitalization.
(61)	Reversal of immune-checkpoint inhibitor fulminant myocarditis using personalized-dose-adjusted abatacept and ruxolitinib: proof of concept	Case Report	1	Ruxolitinib + Abatacept	Patient survived with recovery of cardiac function.
(62)	Abatacept/ruxolitinib and screening for concomitant respiratory muscle failure to mitigate fatality of immune-checkpoint inhibitor myocarditis	Cohort Study	30	Ruxolitinib + Abatacept	Mortality rate in the combination therapy group decreased to 3.4% (historical control ~60%).
(63)	Novel use of abatacept and ruxolitinib as salvage therapy in steroid-refractory immune checkpoint blockade-induced myocarditis with myasthenia and myositis overlap syndrome	Case Report	1	Ruxolitinib (15 mg twice daily) + Abatacept	Clinical symptoms resolved, troponin levels significantly decreased, successfully weaned from ventilator and discharged.
(64)	Tofacitinib for the treatment of immune-related adverse events in cancer immunotherapy: a multi-center observational study	Multi-center Retrospective Cohort Study	48 (Myocarditis subgroup)	Tofacitinib (Primary regimen: 5 mg twice daily)	87.5% (42/48) of myocarditis patients achieved clinical remission.
(65)	JAK inhibitors for the treatment of life-threatening and refractory immune-related adverse events secondary to immune checkpoint inhibitors: a prospective cohort study	Prospective cohort study	29(8 Myocarditis subgroup)	22 Tofacitinib,1 upadacitinib	70.8% complete remission in irAE, 6/8 in myocarditis patients
(66)	Successful Use of Ruxolitinib for Steroid-Refractory Immune Checkpoint Inhibitor-Associated Myocarditis	Case Report	1	Ruxolitinib (Initial 5 mg twice daily, increased to 10 mg twice daily)	Sustained clinical improvement.

Abatacept is a cytotoxic T lymphocyte-associated antigen 4 immunoglobulin fusion protein that binds to CD80/CD86 on the surface of APCs, blocking CD28 co-stimulation and inducing T cell anergy. It has been confirmed as an effective salvage therapy for critical myocarditis, but its onset of action is slow. Ruxolitinib acts on the downstream JAK-STAT inflammatory pathway of T cell activation, directly reducing CD86 protein expression in macrophages, amplifying the target blockade of abatacept, and providing complementary onset times (61). Studies by Joe-Elie Salem et al. have shown that combining the CTLA-4 immune checkpoint inhibitor abatacept with ruxolitinib for the treatment of severe ICI-associated myocarditis, along with early monitoring of respiratory muscle function and mechanical ventilation support, significantly reduced mortality in the combination therapy group (1/30) compared to the group treated as per current expert consensus guidelines (6/10). In the patients with grade 3 or higher myocarditis combined with myositis, the combination therapy was associated with a lower mortality rate of 3.4% (1/22), compared to a higher rate of approximately 80%(6/8) in historical treated patients, demonstrating promising clinical prospects for the combination therapy (62). Of note, interpretation of these encouraging outcomes requires caution. Given concurrent administration of abatacept, ruxolitinib, background glucocorticoids and supportive care, the independent therapeutic effect of JAK-inhibition cannot be reliably isolated. Furthermore, cumulative case evidence corroborates the efficacy of this combination therapy in managing refractory ICIM (61, 67). Beyond established protocols, baricitinib has been reported to alleviate myocardial inflammation in a case of autoimmune myocarditis secondary to STAT1 gain-of-function mutations, although its capacity to reverse pre-existing fibrosis remains limited (68). Mechanistically, preclinical models indicate that baricitinib promotes macrophage polarization toward the M2 phenotype and inhibits the phosphorylation of JAK1 and STAT3, thereby enhancing cardiac performance and attenuating both inflammation and ICI-induced fibrosis (69). Accordingly, we hypothesize that baricitinib holds potential as a future therapeutic option for ICIM.

However, JAK inhibitor therapy is accompanied by related adverse events, such as an increased risk of infection. Common serious infections associated with JAK inhibitors include pneumonia, urinary tract infection, cellulitis, and herpes zoster (70), with a higher incidence of herpes zoster reactivation observed compared to other targeted immunomodulators (71, 72). Long-term use may increase the potential risk of malignancy, particularly skin cancers. Pfizer has warned that, excluding non-melanoma skin cancer, the incidence of malignancies is 1.13 (95% CI 0.94–1.35), with lung cancer being the most common (73). Furthermore, the European Medicines Agency’s Committee for Medicinal Products for Human Use (EMA CHMP) and the U.S. Food and Drug Administration (FDA) have added warnings regarding thrombosis and intestinal perforation to the labeling of baricitinib and tofacitinib. Therefore, the timing of JAK inhibitor administration warrants careful consideration.

To date, clinical documentation of baricitinib in the context of ICIM is absent, with its current utility restricted to autoimmune conditions such as rheumatoid arthritis and alopecia areata. Furthermore, accumulating case evidence indicates that JAK inhibitors have been successfully deployed in the management of ICI-associated nephritis and immune-mediated enterocolitis, highlighting the expansive therapeutic horizon of JAK inhibition across a broad spectrum of irAEs (74, 75).

## 4 Discussion

ICIM, as a severe and potentially fatal immune-related adverse event, has attracted widespread attention in recent years due to its complex pathological mechanisms and multiple challenges in clinical diagnosis and management. The Janus kinase (JAK) signaling pathway, a key regulator of immune-inflammatory responses, plays a central role in the development and progression of this form of myocarditis. Based on this mechanism, JAK inhibitors, as targeted therapeutic agents, demonstrate significant potential in modulating immune responses and alleviating myocardial inflammatory injury, making them a current focus of research.

Treatment options for glucocorticoid-resistant ICIM are limited with existing conventional therapies. Current guidelines have not explicitly recommended the use of tofacitinib, ruxolitinib, or other JAK inhibitors for this condition. Only some retrospective studies and case series report favorable outcomes in ICIM patients treated with JAK inhibitors, particularly ruxolitinib. Currently, direct comparative studies evaluating the efficacy and safety of JAK inhibitors versus other immunosuppressants in ICIM remain lacking. Evidence from immune-mediated inflammatory diseases demonstrates that JAK inhibitors achieve rapid early-onset and superior efficacy in refractory cases, offering a more pronounced glucocorticoid-sparing effect compared with biologic disease-modifying antirheumatic drugs (bDMARDs), though accompanied by higher observed risks of serious infections and herpes zoster (76–78). Furthermore, clinical data from rheumatoid arthritis cohorts indicate a higher reported probability of major adverse cardiovascular events (MACE) and all-cause death (ACD) in JAK inhibitor groups relative to TNF inhibitor group. While long-term JAK inhibitor therapy has been associated with increased rates of malignancies—specifically first and second primary keratinocyte cancers—comparative evidence regarding tumor progression or secondary primary malignancies in oncology patients is not yet established (79–81). Encouragingly, the safety management measures initiated following the Pharmacovigilance Risk Assessment Committee (PRAC) communications have effectively mitigated cardiovascular and related risks (82).

Since most safety warnings for JAK inhibitors are derived from rheumatoid arthritis and other autoimmune-disease cohorts, patients with ICIM and underlying cancer have distinct baseline risks including pre-existing malignancy, ICI-triggered immune perturbation, multi-organ comorbidities and concurrent polypharmacy. Safety findings from non-oncological populations therefore cannot be uncritically extrapolated to this patient subgroup. Moreover, therapeutic JAK inhibition suppresses excessive myocardial inflammatory injury in ICIM, yet the same JAK-STAT axes are indispensable for mounting effective anti-tumor T-cell responses. There exists a potential therapeutic trade-off: robust JAK-blockade may mitigate cardiac immune injury while at least theoretically dampening ICI-driven anti-tumor immunity. Recent pre-clinical single-cell work in a PD-1-blockade-triggered irAE mouse model demonstrates that selective JAK1 inhibition can suppress pathogenic CTL-irAE cells and ameliorate cardiac irAEs without abrogating CTL1-driven anti-tumor immunity. By contrast, broader pan-JAK or JAK2-targeted inhibition impairs anti-tumor T-cell function alongside mitigating tissue immunopathology. Despite these mechanistic insights, real-world clinical data on tumor progression dynamics after JAK-inhibitor treatment in patients with ICIM remain scarce, and subgroup-specific prospective safety and efficacy data are urgently required.

Although these studies provide preliminary evidence supporting the use of JAK inhibitors for ICIM, several critical limitations in the current evidence base warrant caution. First, the available data are predominantly restricted to case reports and small retrospective series. Though small-size prospective cohort study has been published, large-scale randomized controlled trials (RCTs) and multi-institutional prospective cohorts remain absent. This gap is primarily attributable to the low incidence and fulminant clinical course of ICIM, which pose severe logistical and ethical challenges to conducting randomized, placebo-controlled trials in critically ill populations. Additionally, significant heterogeneity in the timing, dosage, and treatment duration of JAK inhibitors, alongside variations in concurrent corticosteroid or immunosuppressive regimens, outcome metrics, and follow-up durations, severely compromises inter-study comparability. Second, publication bias poses a substantial concern. Current literature on JAK inhibitors for ICIM consists almost exclusively of positive findings, whereas treatment failures, lack of efficacy, or severe adverse events are rarely published—a phenomenon compounded by the inherent tendency of small-sample studies to yield positive results. This selective reporting inevitably causes an overestimation of JAK inhibitor efficacy and restricts the generalizability of the findings. Future research should prioritize international multicenter registries to harvest high-quality, real-world data, actively promote the reporting of negative outcomes, and utilize adaptive prospective designs to establish a more robust evaluation of efficacy and safety. The JAK signaling pathway plays a fundamental role in orchestrating the immuno-inflammatory cascade of immune checkpoint inhibitor-associated myocarditis (ICIM), establishing JAK inhibition as a highly promising, innovative therapeutic frontier. Concomitant with the evolution of precision medicine and the continuous refinement of immunomodulatory frameworks, future research must converge on three critical imperatives: 1) validating stratification criteria across multicenter prospective cohorts by leveraging the dynamic kinetics of immunobiomarkers (e.g., serum IFN-γ, IL-6), early cardiotoxicity sentinels (e.g., cTnI, NT-proBNP), and novel metabolomic signatures to achieve precise pre-treatment endotyping; 2) architecting predictive models driven by real-world evidence that integrate baseline clinical phenotypes, tumor histologies, specific ICI regimens, and early immunological trajectories to orchestrate personalized JAK inhibitor selection, optimal initial dosing, tailored tapering schedules, and proactive windows for adverse event mitigation; and 3) deciphering the immunodynamic repercussions of concurrent JAK inhibition and ICI therapy, specifically dissecting whether this dual axis compromises systemic anti-tumor surveillance. Ultimately, bridging the gap between empirical recommendations and scalable clinical practice guidelines relies on high-fidelity data from prospective randomized controlled trials or target trial emulations, which are indispensable to unlocking the definitive paradigm of maximizing therapeutic efficacy while minimizing toxicities in JAK inhibitor-managed ICIM.
