# Supplementary material for: Research advances in Janus kinase inhibitors for immune checkpoint inhibitors-related myocarditis
Source: Front Immunol. 2026 Oct 20;17:1857298. doi: 10.3389/fimmu.2026.1857298 (PMC13728778; doi:10.3389/fimmu.2026.1857298)
Supplement: Supplementary file 1 [file Table1.docx]

Supplementary Table 1 Consensus Criteria of the International Cardiac Oncology Society (2022)

Multifocal inflammatory cell infiltration was observed on endomyocardial biopsy (EMB), with >14 inflammatory cells per high-power field (HPF), accompanied by prominent myocardial cell loss under light microscopy.

Elevated cardiac troponin levels (new-onset or significant changes from baseline), **plus fulfillment of either 1 major criterion or 2 minor criteria** (with simultaneous exclusion of acute coronary syndrome and acute infectious diseases).

**Major Criteria:**

Cardiac Magnetic Resonance (CMR) findings consistent with the diagnosis of acute myocarditis (modified Lake Louise Criteria)

**Minor Criteria:**

1. Clinical syndrome (including any of the following: fatigue, myalgia, chest pain, diplopia, ptosis, dyspnea, orthopnea, lower extremity edema, palpitations, dizziness or vertigo, syncope, muscle weakness, cardiogenic shock)

2. Ventricular arrhythmias and/or new-onset conduction system disorders

3. Decreased cardiac function (with or without segmental wall motion abnormalities showing a non-stress cardiomyopathy pattern)

4. Other immune-related adverse events, particularly myositis, myopathy, and myasthenia gravis
